# Dr. Fessenden Nott Otis

**Published:** 1900-06

**Authors:** 


DR. FESSENDEN NOTT OTIS, of New York, died at New Orleans, La., Thursday, May 24, 1900, aged 75 years.

Dr. Otis was born on May 6, 1825, in Ballston, N.Y. He was educated at Fairfield and Amsterdam, N.Y. He later studied medicine at the New York University and the New York Medical College. He was graduated from the latter institution in 1852. He was resident assistant physician at Blackwell's Island Hospital in 1852 and 1853. He then became surgeon to the United States Mail Steamship Company and remained with that company until 1860. In the following year he was appointed surgeon of the police department, and served in that capacity until 1872. From 1870 to 1872 he was president of the board of police surgeons and from 1869 to 1873 he was superintending surgeon to the Pacific Mail Steamship Company.

In 1862 he was appointed attending physician to the Demilt Dispensary, and continued as such until 1870. He later became surgeon to the Strangers' Hospital and president of its medical board. In 1862 he was chosen clinical lecturer at the College of Physicians and Surgeons, and nine years later became a clinical professor there. Since 1874 he had been consulting surgeon to various hospitals. In 1872 he was president of the American Dermatological Society. He was the inventor of a number of medical instruments, and was the author of several works on medicine and other subjects. The titles of some of his books are, Lessons in Drawing, Studies of Animals and Landscapes; Tropical Journeyings, and History of the Panama Railroad and its Commercial Connections.

